# Carbon Fiber Implants in Orthopaedic Oncology

**DOI:** 10.3390/jcm11174959

**Published:** 2022-08-24

**Authors:** Caleb M. Yeung, Abhiram R. Bhashyam, Shalin S. Patel, Eduardo Ortiz-Cruz, Santiago A. Lozano-Calderón

**Affiliations:** 1 Division of Orthopaedic Oncology, Department of Orthopaedic Surgery, Massachusetts General Hospital, Harvard Medical School, Boston, MA 02144, USA; 2 Division of Orthopaedic Oncology, La Paz University Hospital, MD Anderson Cancer, 28033 Madrid, Spain

**Keywords:** carbon fiber, orthopaedic oncology, prophylactic fixation, pathologic fracture, radiation therapy

## Abstract

Carbon fiber offers numerous material benefits including reduced wear, high strength-to-weight ratio, a similar elastic modulus to that of bone, and high biocompatibility. Carbon fiber implants are increasingly used in multiple arenas within orthopaedic surgery, including spine, trauma, arthroplasty, and oncology. In the orthopaedic oncologic population, the radiolucency of carbon fiber facilitates post-operative imaging for tumor surveillance or recurrence, the monitoring of bony healing and union, and radiation mapping and delivery.

## 1. Introduction

Orthopaedic surgery and materials science are closely intertwined, as the success of many orthopaedic devices are contingent on the material properties of the components used. Specifically in orthopaedic oncology, advancements in implant materials and design are crucial to preservation of function and quality of life in oncology patients after the tumor resection or treatment of metastatic disease. Reconstructive implants in orthopaedic oncology ideally maintain limb function and bone strength, allow for full bone healing, minimize risk of infection and implant failure, and facilitate the specific needs of oncology patients, including surveillance imaging, the visualization of bony union or healing, and radiation mapping and delivery. Historically, metallic implants, frequently used for such reconstructive purposes, have been associated with the tradeoff of imaging artifact. Carbon fiber has more recently been making inroads within orthopaedic surgery and oncology, as its material properties have particular advantages in comparison to metallic materials. As a material, its benefits have long been recognized and applied to numerous industrial needs, ranging from aerospace to civil engineering [1]. Within orthopedic oncology, carbon fiber implants have demonstrated promising applications as a material for implants utilized for pathologic fracture fixation or reconstruction. In this article, we review the material strengths of carbon fiber and the literature concerning its current applications in orthopaedics so far. We also present a series of case examples that demonstrate the benefits of carbon fiber implants specific to the orthopaedic oncologic population.

## 2. Material Properties and Benefits of Carbon Fiber

Carbon fiber was first discovered in 1860 and used for light bulb filaments, which typically involved baking cotton threads or bamboo slivers at high temperatures to carbonize them. This was followed by the development of high-performance carbon fibers by using either rayon or polyacrylonitrile as the material to be carbonized, yielding carbon fibers with very high tensile strength and elasticity, while maintaining a high strength-to-weight ratio. Iterative improvements in the production process for carbon fiber, as well as the incorporation of carbon fiber into composite materials, led to widespread commercial application, including use in sporting goods, aerospace applications such as in heat shields or aircraft brakes, aircraft frames such as in sailplanes or military aircraft, race cars, and structural reinforcement of structures such as bridges. Perhaps the most high profile example of a carbon fiber product is the Boeing 787 aircraft, whose structure is 50% carbon fiber [2].

When employed in composite materials within the medical domain, carbon fiber is typically combined with several types of resin matrices, one of the most common of which is carbon fiber–polyether ether ketone (CF–PEEK). There are numerous properties of carbon fiber that make it ideal for orthopaedic applications. It is highly biocompatible and chemically inert [3], generating no cellular toxicity in in vitro studies [4] and only a non-specific foreign body reaction in animal studies [5]. Its elastic modulus, a measure of resistance to deformity under stress, is close to that of bone, an important advantage over other implant materials [6]. The estimated elastic modulus of carbon fiber is 3.5 gigapascals (GPa); cortical bone has an elastic modulus of 12–20 GPa and cancellous bone 1 GPa. By contrast, the elastic modulus of stainless steel is 230 GPa and titanium ranges from 106–155 GPa [7,8,9]. The similar elastic modulus of carbon fiber implants to bone helps to lessen stress concentration at the bone–implant interface [10,11], though it is important to note that additional studies are needed to better validate whether this allows for improved healing potential, or may simply translate to insufficient stiffness for healing in certain unstable fracture types in the trauma setting [12,13]. Importantly, in this regard, the modulus of carbon fiber can be adjusted in manufacturing to match either cortical or cancellous bone [14].

The ability to withstand fatigue strain is yet another benefit of carbon fiber implants. Traditional implants demonstrate higher failure rates, especially in pathologic fractures, often due to non-union or hardware failure [15]. By contrast, CF–PEEK demonstrates the ability to withstand high strain loading, up to one million loading cycles, without evidence of failure [16]. The interface wear characteristics of carbon fiber are similarly encouraging. One study simulating a total hip arthroplasty investigated the wear results of a ceramic head on a CF–PEEK cup, and demonstrated a volumetric wear rate of 0.3 mm^3^/Mc (million cycles), lower than that of ceramic on cross-linked, ultra-high-molecular-weight polyethylene (UHMWPE), metal on cross-linked UHMWPE, or ceramic or metal on conventional UHMWPE [17]. This is particularly important when considering potential toxicity or allergic reactions from wear particles [18,19]. As a relatively more recent material used in the orthopaedic setting, there are relatively fewer large, long-term studies concerning carbon fiber implants compared to their metallic counterparts, but their durability has appeared promising thus far in the literature [20].

One of the most important advantages of carbon fiber implants over metallic implants is their radiolucency. On both magnetic resonance imaging (MRI) and computed tomography (CT), carbon fiber has minimal scatter or susceptibility artifact, respectively [21]. This radiolucency allows for improved post-operative monitoring of fracture healing and surveillance for local disease recurrence or progression in the orthopaedic oncologic population. In a comparative study comparing MRI signal loss in patients with femoral or tibial CF–PEEK or titanium implants, CF–PEEK implants demonstrated substantially less signal loss and MRI susceptibility artifact than titanium nails. Visualization scores, as graded by a musculoskeletal radiologist, were significantly higher in the CF–PEEK group across all MRI sequences, including T1-weighted, short tau inversion recovery (STIR), and contrast-enhanced, T1-weighted, fat-saturated sequences [21]. Additionally, many orthopaedic oncology patients require post-operative radiotherapy. The artifact generated by conventional metallic implants often interferes not only with mapping for radiation planning, but also with accurate dose calculation and delivery [22,23,24]. Indeed, as radiation planning frequently relies on the use of high-quality advanced imaging modalities such as CT or MRI to plan radiation therapy doses and distribution, metal implants hinder this by both creating imaging artifact, increasing target volume, and often requiring potentially erroneous assumptions to be made about the degree of absorption by the implant [25].

**Figure 1:**
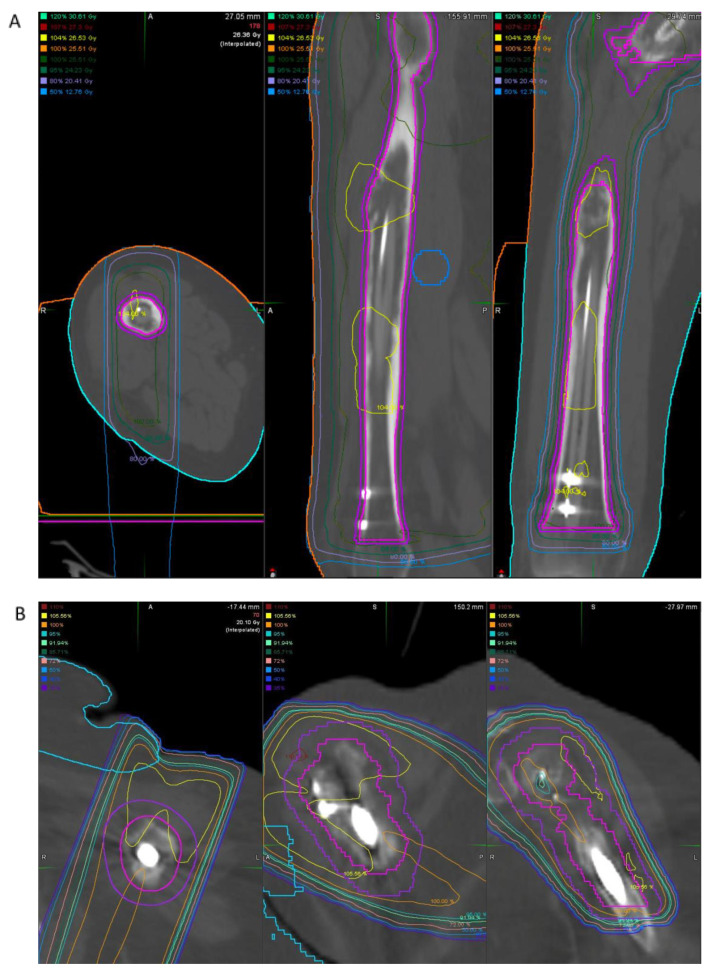
(**A**) Radiation therapy plan for a patient with impending pathologic fracture of the femur status post-intramedullary fixation with carbon fiber nail. Note is made of the minimal artifact from the intramedullary nail and relatively small amount of artifact from the metallic interlock screws. (**B**) Radiation therapy plan for a patient with impending pathologic fracture of the humerus status post-intramedullary fixation with titanium nail. Note the increased artifact caused by the intramedullary nail and interlock screws leading to increased target volume.

Figure 1 illustrates an example of reduced target volume during radiation planning in a patient with a carbon fiber intramedullary nail (Figure 1A) compared to a patient with a conventional metallic nail (Figure 1B). This reduced target volume is facilitated by the reduced artifact of the carbon fiber nail on CT imaging. 

With regard to CT imaging, carbon fiber demonstrates similar Hounsfield units compared to biological material, allowing for more accurate dose calculations [26]. In one large study, the volume of artifact created by carbon fiber pedicle screws on CT imaging was significantly less than that of titanium screws (494 ± 246 mm^3^ compared to 937 ± 322 mm^3^, respectively) [26]. As might be expected, the reduced artifact generated by carbon fiber compared to metallic implants during imaging for radiation mapping translates to improved radiation delivery [27] and reduced dose perturbations [26,28]. In a laboratory model, CF–PEEK nails allowed for more efficient radiation delivery to a target volume compared to titanium nails, and allow for more accurate radiation dosing, with smoother dose distributions [29]. Further data are notably required, however, on the impact of radiation on the material properties of carbon fiber. 

## 3. Carbon Fiber Implants in Orthopaedic Surgery

Given the numerous material strengths of carbon fiber, it is no surprise that their use in orthopaedic implants has been increasingly studied across nearly all orthopaedic subspecialties in multiple applications. Since the 1990s, carbon fiber implants have been used for spinal surgical procedures such as posterior lumbar interbody fusion [30] and anterior cervical discectomy and fusion, demonstrating low rates of complications and specifically implant-related complications, as well as favorable outcomes [31]. They have also been employed in cages used for spinal column reconstruction in patients undergoing thoracolumbar corpectomies, again demonstrating promising long-term results with regards to cage durability, facilitation of radiographic monitoring of fusion, and low complication rates [32].

Orthopedic trauma surgery has more recently explored the use of carbon fiber plates in a variety of applications, including intramedullary nailing, proximal humerus or distal radius plating, and dynamic compression plating. When compared to their corresponding metallic implant counterparts, carbon fiber implants have been found to have similar performance in four-point bending, torsional, and bending fatigue, as well as reduced particle generation in wear testing [16]. Clinically, these plates have demonstrated promising outcomes as well. One retrospective review of 71 distal radial fractures treated with CF–PEEK plates found satisfactory post-operative functional scores according to the modified Mayo wrist score, excellent radiographic results, and only one device complication due to aseptic loosening [33]. Paralleling the burgeoning application of carbon fiber implants in trauma, it has also been employed in arthroplasty applications, with favorable early results [34,35,36].

Carbon fiber implants have promising applications particularly with regard to spinal tumor and spinal metastatic surgery. A large comparison of 78 patients with spinal metastases who underwent fixation with CF–PEEK versus titanium implants noted similar rates of post-operative clinical complications and hardware failure. Of note, the CF–PEEK group had longer operative times and higher blood loss, which might suggest a learning curve with these newer implants [37]. Another study of 22 patients with primary spine tumors who underwent surgery using CF–PEEK implants demonstrated similar outcomes to titanium implants, but noted higher ease with radiation planning and administration in these patients [23]. 

Specifically with regard to radiotherapy in the spinal oncologic setting, precise radiation planning and delivery is critical to avoid damage to the neural structures in close proximity. In simulations utilizing carbon-fiber-reinforced polyether ether ketone and titanium implants (CFP–T), CFP–T implants allowed the complete visualization of the spinal cord on MRI and allowed for minimal differences in radiation dose between planned and measured dose distributions [38].

## 4. Case Examples

In this section, we present five cases from our department utilizing carbon fiber implants to highlight their unique advantages in the treatment of orthopaedic oncology patients.

### 4.1. Case Example 1–Prophylactic Fixation via Intramedullary Nailing following Resection of Cortical Margin for Myxoid Liposarcoma Resection

**Figure 2:**
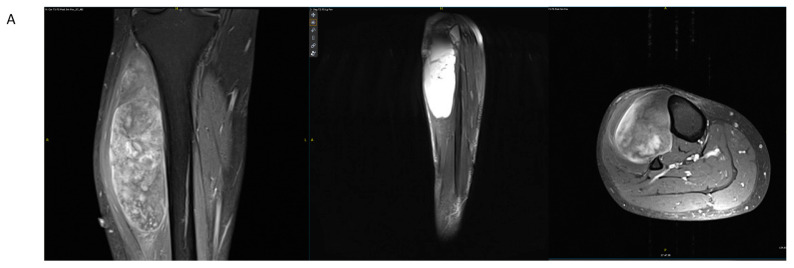

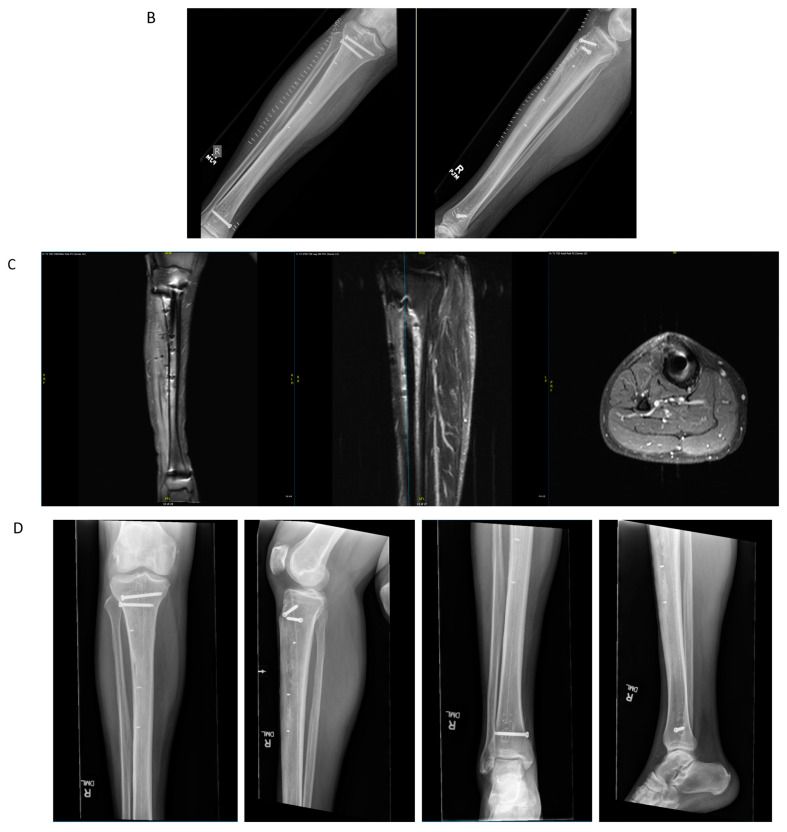
(**A**) Pre-operative T1 post-contrast, fat-saturation coronal; T2 fat-suppression sagittal; and T1 post-contrast, fat-saturation axial magnetic resonance images of a right tibia demonstrating a myxoid liposarcoma. (**B**) Post-operative radiographs demonstrating interval placement of an intramedullary carbon fiber nail. Note is made of a cortical defect in the tibia to allow for adequate resection margins; as such, a carbon fiber was placed for fracture prophylaxis. (**C**) The 9-month post-operative T1 post-contrast, fat-saturation coronal; T2 fat-suppression sagittal; and T1 post-contrast, fat-saturation axial magnetic resonance images demonstrating interval resection of the myxoid liposarcoma. Reduced artifact from carbon fiber nail facilitated visualization of the resection bed for monitoring of local recurrence. (**D**) The 20-month post-operative radiographs of the right tibia with intramedullary carbon fiber nail in place and healed cortical defect from prior resection margin.

We present a case of an otherwise healthy 54-year-old male who was referred to our center for management of a progressively enlarging, palpable, non-tender mass on his anterolateral leg. The pre-operative MRI demonstrated a large, heterogeneously enhancing, solid soft-tissue mass that was T1 hypointense and T2 hyperintense (Figure 2A). The mass was located primarily within the tibialis anterior muscle belly in the anterior compartment, and abutted both the tibial and fibular cortices without intramedullary involvement. The neurovascular bundle was displaced but not encased by the mass. A CT-guided percutaneous biopsy was performed. Pathology demonstrated an intermediate-grade myxoid liposarcoma. Staging PET–CT did not reveal any evidence of metastatic disease. The patient underwent a total of 50.4 Gray (Gy) of neoadjuvant photon radiation over 28 fractions, and completed four cycles of neoadjuvant doxorubicin–ifosfamide–mesna chemotherapy.

The patient subsequently underwent wide excision. In order to maximize negative margins, a partial corticectomy of the anterolateral tibia was performed. A carbon fiber intramedullary tibial nail was prophylactically placed in order to prevent fracture after cortical resection (Figure 2B). Post-operative surveillance MRI demonstrated the resection of the myxoid liposarcoma with minimal artifact, facilitating monitoring of local recurrence (Figure 2C). Three cycles of adjuvant chemotherapy were completed. The patient remains disease-free at 2 years of follow-up. Visualization of disease recurrence is facilitated by the reduced imaging artifact from the carbon fiber nail. The 20-month follow-up radiographs are shown in Figure 2D.

### 4.2. Case Example 2—Plate Fixation of Medial Femoral Condyle Osteoarticular Allograft following Medial Femoral Condyle Resection of Chondrosarcoma

**Figure 3:**
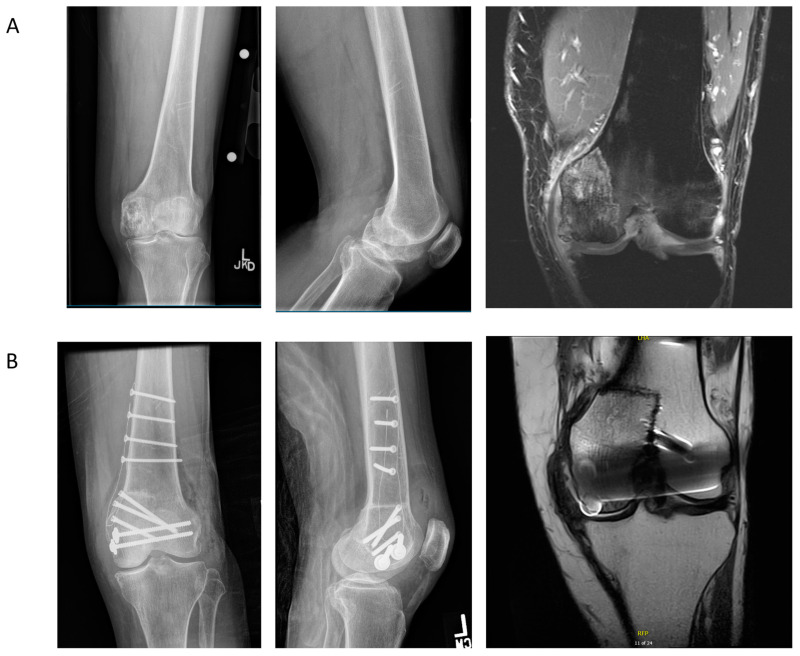
(**A**) Pre-operative anteroposterior (AP) and lateral radiographs of the distal femur and T1-post-contrast fat-saturation coronal magnetic resonance (MR) image of the knee, demonstrating a grade 2 chondrosarcoma of the medial femoral condyle. (**B**) The 12-month post-operative AP and lateral radiographs and proton density coronal MR image of the knee, demonstrating interval hemicondylar resection and osteoarticular allograft reconstruction with a carbon fiber plate and supplemental screw fixation. Minimal susceptibility artifact from the plate is noted with comparatively higher artifact from the metallic screws.

We present a case of a 63-year-old female who presented for evaluation of medial-sided left knee pain. The patient had a history of a low-grade cartilaginous neoplasm, treated eight years prior with intralesional curettage and bone grafting at an outside institution. No surgical adjuvants were used during the index operation. She continued to have medial-sided knee pain, and three years after her index operation, underwent knee arthroscopy and medial meniscectomy for a meniscal tear. Surveillance radiographs performed by her prior surgeon demonstrated concerning changes in the medial femoral condyle that were further assessed on MRI (Figure 3A). Percutaneous CT-guided biopsy at our institution demonstrated grade 2 chondrosarcoma. Staging CT of the chest demonstrated no pulmonary metastases, and bone scan revealed disease localized to the left medial femoral condyle. The patient underwent wide excision of the medial femoral condyle with osteoarticular allograft reconstruction using a carbon fiber plate and supplemental screw fixation (Figure 3B). Substantially reduced susceptibility artifact is noted on the post-operative MRI, allowing for monitoring of local disease recurrence. The patient had no evidence of local recurrence at 12-month follow-up.

### 4.3. Case Example 3—Plate and Intramedullary Nail Fixation of Allograft Reconstruction of Femoral Intercalary Resection of Chondrosarcoma

**Figure 4:**
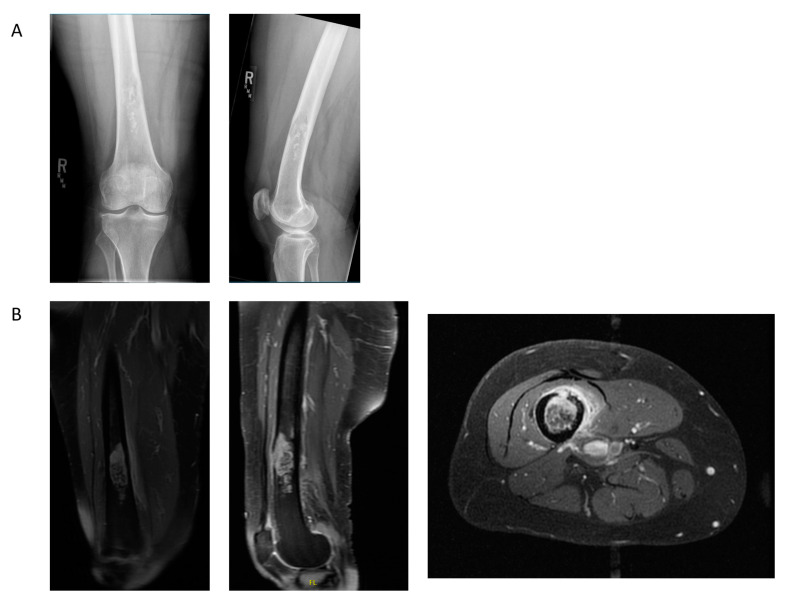

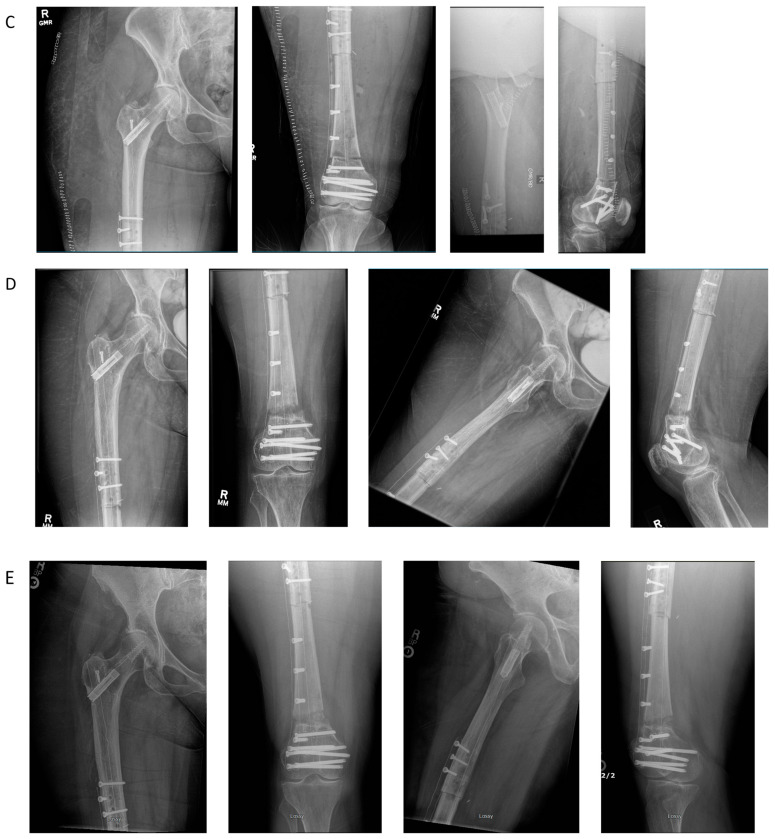
(**A**) Pre-operative anteroposterior (AP) and lateral radiographs of the right distal femur, demonstrating a lesion in the distal metadiaphysis. (**B**) Pre-operative post-contrast, T1 fat-suppressed coronal, sagittal, and axial magnetic resonance (MR) images of the distal femur further characterizing the high-grade dedifferentiated chondrosarcoma. Note is made of the cortical breach and extramedullary soft-tissue component. (**C**) Immediate post-operative AP and lateral radiographs of the right femur, demstatus post intercalary resection with allograft reconstruction utilizing a carbon fiber cephalomedullary nail and carbon fiber plate. (**D**) 15-month post-operative AP and lateral radiographs of the right femur, demonstrating allograft non-union and hardware loosening. (**E**) The 6-week post-operative AP and lateral radiographs of the right femur status-post removal of the carbon fiber plate and non-union revision open reduction internal fixation with iliac crest bone graft with use of a carbon fiber plate. The carbon fiber hardware facilitated clearer visualization of the allograft–native bone interface to assess for subsequent bony union.

We present a case of an otherwise healthy 57-year-old female with right thigh pain for three months. Plain films (Figure 4A) and magnetic resonance (MR) images (Figure 4B) demonstrated findings concerning for a malignant chondroid neoplasm in the right femur distal metadiaphysis. Staging CT of the chest was negative for pulmonary metastases, and whole-body bone scan demonstrated no other sites of disease. The patient underwent intercalary resection with allograft reconstruction using a carbon fiber cephalomedullary nail and carbon fiber plate (Figure 4C). Pathology of the excised specimen demonstrated grade 3 dedifferentiated chondrosarcoma with high-grade osteosarcomatous differentiation and negative surgical margins. The patient completed five cycles of adjuvant chemotherapy consisting of cisplatin and doxorubicin with pegfilgrastim support. At her 15-month follow-up appointment, the patient reported difficulty with ambulation without an assistive device. Plain films at that time demonstrated a lack of bony healing at the distal allograft–native bone interface and hardware loosening (Figure 4D). Her non-union was more easily appreciable on plain radiographs due to the radiolucency of the carbon fiber implants. The patient subsequently underwent removal of hardware and revision open reduction internal fixation with iliac crest bone graft, which was tolerated well. Six-week post-operative radiographs demonstrated expected early findings without evidence of hardware complication (Figure 4E). 

### 4.4. Case Example 4—Plate Fixation of Allograft Reconstruction of Proximal Humerus following Intercalary Resection of Parosteal Myositis Ossificans

**Figure 5:**
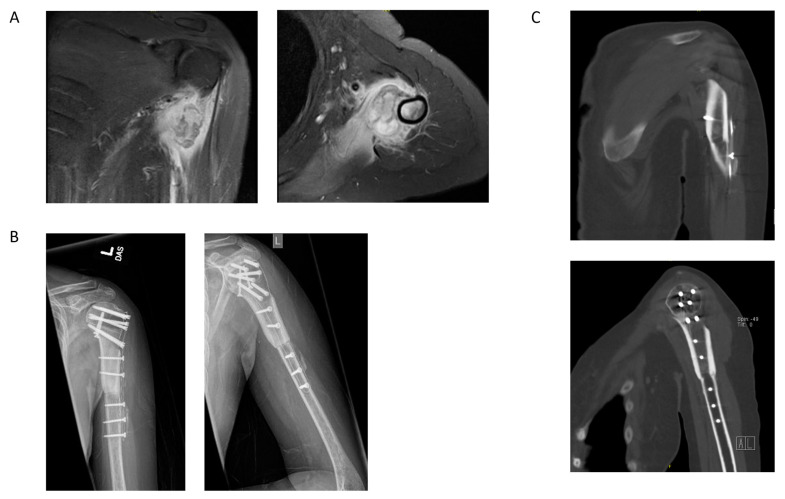
(**A**) Pre-operative T1 post-contrast, fat-saturation coronal and axial magnetic resonance (MR) images showing a large, enhancing tissue mass on the left proximal humerus without marrow involvement. (**B**) The 1-month post-operative AP and lateral radiographs following left humerus intercalary resection and allograft reconstruction with carbon fiber plate and cortical screws demonstrating incomplete fusion between the allograft and the native humerus. (**C**) The 8-month post-operative coronal and sagittal computed tomography (CT) images showing partial fusion. The carbon fiber plate facilitated visualization of the site of allograft and native humerus fusion status.

We present a case of a 36-year-old female with no past medical history who presented with 3 months of left shoulder pain, paresthesias, and pain with shoulder movement. She previously received a steroid injection to the shoulder without significant relief. Biopsy conducted at an outside institution was concerning for high-grade osteosarcoma, and she was referred to our care. MR images (Figure 5A) demonstrated an exophytic mass of the proximal medial humerus. Staging CT chest showed no intrathoracic metastatic disease. The presumed pre-operative diagnosis was high-grade surface osteosarcoma with intramedullary extension. She underwent intercalary resection with allograft reconstruction using a carbon fiber proximal humerus plate. Final pathology of the resected mass revealed parosteal myositis ossificans. The 1-month post-operative radiographs (Figure 5B) demonstrated an intact carbon fiber plate and screws, with incomplete fusion of the allograft and native humerus. Computed tomography at 8 months post-operatively (Figure 5C) showed partial, but improved, fusion status at present. The carbon fiber plate facilitated visualization of the status of her fusion at the allograft and native humerus interface.

### 4.5. Case Example 5—Revision Plate and Nail Fixation with Fibular Autograft Augmentation of Prior Tibial Intercalary Allograft Failure

**Figure 6:**
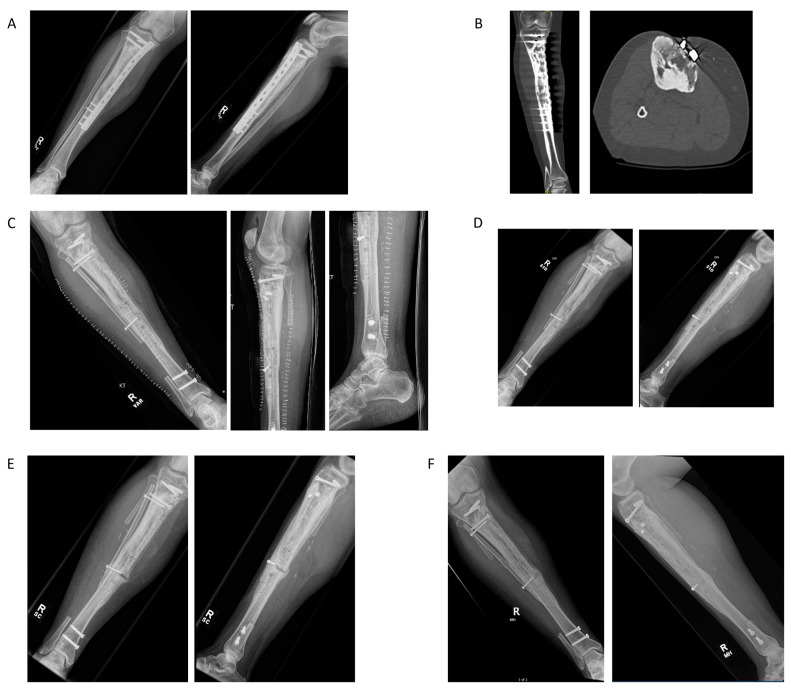
(**A**) Pre-operative AP and lateral radiographs demonstrating prior segmental resection of the proximal right tibia with allograft secured with a medial plate and multiple screws. (**B**) Pre-operative coronal and axial computed tomography (CT) images demonstrating potential hardware failure and concern for allograft fracture. (**C**) Immediate post-operative AP and lateral radiographs showing carbon fiber intramedullary rod in place and transposed vascularized fibular autograft secured with two screws. Note is made of one broken screw fragment from her prior operation that was retained in the proximal tibia. (**D**) AP and lateral radiographs 4-months post-operatively demonstrating a new unicortical allograft fracture after mechanical fall. (**E**) AP and lateral radiographs 8-months post-operatively bridging callus at the site of the mid-tibial unicortical allograft fracture. (**F**) AP and lateral radiographs 2-years post-operatively showing healed mid-tibial unicortical allograft fracture.

We present the case of a 41-year-old female with a past medical history of heart failure, endometriosis, and hypothyroidism, who was previously treated at the age of 14 for osteosarcoma of the right proximal tibia and underwent intercalary allograft reconstruction. She presented to our care with allograft failure. With regard to her oncologic history, she initially presented to care at the age of 14 with 3–4 months of progressive swelling below the right knee. She underwent an open biopsy, which revealed high-grade osteosarcoma; she had a negative metastatic work-up. She underwent neoadjuvant and adjuvant chemotherapy with cisplatin and doxorubicin alternating with high-dose methotrexate, and bleomycin/cytoxan/dacarbazine. She then underwent intercalary resection of the right tibia with allograft reconstruction using conventional metallic plate and screw fixation (Figure 6A). Her post-operative course was complicated by right tibial abscess requiring incision and drainage and muscle flap and skin graft to the right tibia. She remained disease-free for 23 years.

She presented to our care with increasing pain and decreased ability to bear weight in the right lower extremity. She had normal inflammatory markers and normal serum white blood cell count. Radiographs and CT scan imaging were concerning for allograft failure (Figure 6B). A subsequent bone scan demonstrated increased signal at the proximal bone–implant interface. She underwent surgical exploration and hardware removal. Three screws were noted to be broken, two of which were removed; the plate was substantially loose proximally. Augmentation with a vascularized fibular autograft transposed to the tibia and prophylactic fixation with a tibial carbon fiber nail was performed (Figure 6C). She did well post-operatively.

4 months after surgery, she sustained a mechanical fall. A unicortical fracture at the allograft and host bone interface was identified on radiographs without evidence of hardware complication (Figure 6D). Her fracture was managed non-operatively. Subsequent radiographs demonstrated progressive healing at the fracture site, with visualization facilitated by the radiolucency of the carbon fiber implant (Figure 6E). Radiographs taken 2-years post-operatively demonstrated complete healing of the fracture, with no evidence of hardware complication (Figure 6F). The use of the carbon fiber radiolucent nail facilitated diagnosis of her initial fracture as well as the subsequent monitoring of fracture healing.

## 5. Considerations of Carbon Fiber in Orthopedic Oncology

In these cases, carbon fiber implants were applied for an range of applications in orthopaedic oncology patients with examples of the radiologic properties of carbon fiber facilitating post-operative surveillance of tumor recurrence and bony healing. Although preliminary studies in the orthopaedic oncologic population have demonstrated promising early results [39], future studies are needed to determine the clinical outcomes of orthopaedic oncologic patients undergoing reconstruction or fixation with these implants, with additional investigation into the purported benefits of carbon fiber as it pertains to reducing stress concentration, enhancing accuracy of radiotherapy, and tracking implant failure rates. 

There are several important factors that must be considered in the use of carbon fiber implants in orthopaedics. Firstly, in comparison to metallic implants, carbon fiber has a lower load to failure, suggesting titanium or steel may carry a lower risk of implant failure in patients expected to have a high functional status post-operatively [40]. Secondly, unlike metallic implants, carbon fiber implants cannot be bent or contoured intra-operatively. Therefore, surgeons must precisely pre-operatively plan to ensure good implant fit. Thirdly, while the radiolucency of carbon fiber is certainly advantageous for imaging studies post-operatively, intra-operatively it may prove challenging to confirm implant position. With regard to radiation planning, screws for carbon fiber plate fixation or interlock screws are metallic, which still does lead to some imaging artifact.

An additional consideration is the potential for undetected implant failure given the radiolucency of carbon fiber implants, which has been reported in case studies [41]. Finally, other considerations include the higher costs for carbon fiber implants and decreased availability, though these factors are both subject to change with time and increasing usage [42]. 

## 6. Conclusions

Carbon fiber implants have numerous promising advantages for use in orthopaedic oncology, including favorable material and radiologic properties. Additional studies are needed as these implants are more widely utilized in the orthopaedic oncologic population to better characterize long-term implant survival and complications as well as the clinical outcomes associated with their use.

## Data Availability

Not applicable.
